# Development of Noninvasive Classification Methods for Different Roasting Degrees of Coffee Beans Using Hyperspectral Imaging

**DOI:** 10.3390/s18041259

**Published:** 2018-04-19

**Authors:** Bingquan Chu, Keqiang Yu, Yanru Zhao, Yong He

**Affiliations:** 1 School of Biological and Chemical Engineering/School of Light Industry, Zhejiang Provincial Key Laboratory of Chemical and Biological Processing Technology of Farm Products, Zhejiang Provincial Collaborative Innovation Center of Agricultural Biological Resources Biochemical Manufacturing, Zhejiang University of Science and Technology, Hangzhou 310023, China; bqchu@zust.edu.cn; 2 College of Mechanical and Electronic Engineering, Northwest A&F University, Yangling 712100, China; yuke406336022@gmail.com; 3 College of Biosystems Engineering and Food Science, Zhejiang University, Hangzhou 310058, China; xiaoru19863804@sina.com; 4 Key Laboratory of Spectroscopy Sensing, Ministry of Agriculture, Hangzhou 310058, China

**Keywords:** coffee bean, roasting degree, qualitative properties, hyperspectral imaging, chemometric methods

## Abstract

This study aimed to develop an approach for quickly and noninvasively differentiating the roasting degrees of coffee beans using hyperspectral imaging (HSI). The qualitative properties of seven roasting degrees of coffee beans (unroasted, light, moderately light, light medium, medium, moderately dark, and dark) were assayed, including moisture, crude fat, trigonelline, chlorogenic acid, and caffeine contents. These properties were influenced greatly by the respective roasting degree. Their hyperspectral images (874–1734 nm) were collected using a hyperspectral reflectance imaging system. The spectra of the regions of interest were manually extracted from the HSI images. Then, principal components analysis was employed to compress the spectral data and select the optimal wavelengths based on loading weight analysis. Meanwhile, the random frog (RF) methodology and the successive projections algorithm were also adopted to pick effective wavelengths from the spectral data. Finally, least squares support vector machine (LS-SVM) was utilized to establish discriminative models using spectral reflectance and corresponding labeled classes for each degree of roast sample. The results showed that the LS-SVM model, established by the RF selecting method, with eight wavelengths performed very well, achieving an overall classification accuracy of 90.30%. In conclusion, HSI was illustrated as a potential technique for noninvasively classifying the roasting degrees of coffee beans and might have an important application for the development of nondestructive, real-time, and portable sensors to monitor the roasting process of coffee beans.

## 1. Introduction

Coffee (*Coffea*) is one of the most desirable and frequently consumed beverages in the world due to its unique flavor and functional properties. Roasting is an essential step in coffee processing. The desired aroma, flavor, and color of coffee beans are enhanced by roasting, and roasting causes profound changes in the physics, structure, chemical composition, and biological activities of the coffee bean, which can be characterized by the roasting degree (RD) [1]. Coffee beans are roasted using a conventional roasting process at temperatures ranging from 200 °C to 300 °C for 12–20 min [2], and the external color (varying from light to dark brown), the flavor, the amount of dry matter, and the chemical composition of the beans will change with the roasting temperature and time. Over-roasting, inhomogeneous roasting conditions, or other operating errors will produce defective coffee beans, leading to scorching, baking, or cracking [3].

The relationship between the roasting degree and the qualitative properties/bioactive capacity of coffee beans has been reported on by many studies [4,5]. In quantitative terms, alterations in major chemical composition have been described for chlorogenic acid (CA), trigonelline (TG), lipid, sucrose, and amino acids [6,7]. The study by Cho et al. (2017) showed that the CA and TG contents in roasted beans were affected significantly by the roasting conditions. Over-roasted coffee beans (210 °C for 210 s after the first crack) had only 1/5 and 1/3 of the CA and TG contents, respectively, compared with those of under-roasted beans (210 °C for 40 s after the first crack) [8]. Alessandrini et al. (2008) reported that the color of roasted coffee became progressively more brownish and uniform among samples with increased roasting time, which had a direct impact on the density and moisture of the beans [9]. In their study, Hečimović et al. (2011) discussed the fact that the contents of bioactive compounds and the antioxidant capacity of coffee beans varied greatly depending on the respective roasting degree [10]. Those reports indicate that the quality and chemical composition of coffee beans are strictly related to the roasting degree, which is mainly controlled by roasting time and temperature. For this reason, it is important to distinguish defective coffee beans caused by inhomogeneous roasting conditions from coffee beans of the optimum roasting degree during the production of coffee to avoid undesired quality.

Conventionally, the evaluation of roasting degree is performed by one of the following methods: the analysis of physical quality, such as deviations in size, color, weight loss, density, moisture, and flavor; the organoleptic assessment of senses by professional coffee tasters; the measurement of the light reflectance of ground beans; or just judging the color of the beans by sight. Among those, the first three are the handiest variables and are used worldwide to evaluate coffee at the end of the roasting process [9]. Despite their reliability, these methods may be fairly elaborate, costly, time-consuming, or require complex sample preparation, and they are not able to provide real-time, online results, which limits online quality determination during processing. Moreover, some studies have reported that the determination of the light reflectance of ground beans has been proven ineffective, because different roasting degrees of coffee beans can present the same average readings in light reflectance measurements [11]. Thus, there is a need for a rapid, low-cost, reliable, and reproducible analytical method as an alternative tool for the real-time assessment of roasting degree.

Hyperspectral imaging (HSI, also called imaging spectroscopy or imaging spectrometry) is a highly recommended technique that can be applied to obtain an understanding of chemical images. It has the advantage of comprehending the heterogeneity of materials, such as food and agricultural products, for the promotion of quality in processing [12,13,14]. HSI is the integration of spectral analysis and image processing technology; it can simultaneously detect internal composition and spatial distribution information and obtain the distribution of nonhomogeneous properties of an object, as well as monitor particular chemicals using near-infrared rays [15]. On the other hand, because of the complexities of food samples and the tediousness of extracting entire hyperspectral data, it is especially important to choose appropriate exploratory data analysis techniques and chemometric methods for processing hyperspectral data, qualitative information, or quantitative information [14].

Recently, there have been some studies that have investigated the identification of qualitative properties and classification methods in various coffee beans using spectroscopy imaging technology. Caporaso et al. (2018) developed HSI-based quantitative prediction models using partial least squares regression (PLSR) to visualize fat and moisture distribution within individual coffee beans [16]. Zhang et al. (2017) reported that the combination of HSI and chemometric approaches (PLSR and random frog) could be used to characterize the caffeine content of coffee beans [17]. Nansen et al. (2016) found that the quantifiable variables of coffee beans might be predicted accurately based on reflectance features of HSI, which enables deployment of automated machine vision technologies [18]. However, to our best knowledge, few studies concerning nondestructive study of the dynamic variation of the roasting degree of coffee beans in the roasting process have been reported. Therefore, the main focus of this study is to develop an approach for quickly and noninvasively classifying the roasting degree of coffee beans using HSI. The objectives were as follows:

(1) The establishment of a near-infrared (NIR) HSI system in the spectral range of 874–1734 nm to acquire the HSI images of coffee beans during the roasting process;
(2) The extraction of spectral information and selection of effective wavelengths based on chemometric methods for discriminating between the roasting degrees of coffee beans during the roasting process; and
(3) The development of an optimal calibration model for identifying the roasting degree of coffee beans.

## 2. Materials and Methods

### 2.1. Coffee Bean Samples

Columbian Arabica green coffee (*Coffea arabica*) beans were purchased from Qingdao Lefei Trading Co. LTD of China in 2017. Sorted coffee beans of similar shape and size were roasted in batches using a drum roaster (SCR-300, Barwell Corp., Shanghai, China). Seven roasting conditions were selected to produce different roasting degrees of coffee beans, as shown in Table 1. The final temperature of the coffee beans was continuously monitored using an infrared thermoscope (830S1, Testo SE & Co. KGaA, Lenzkirch, Germany). Roasting degrees were determined in three replicates by using a coffee roast analyzer (E20CP, Agtron, Reno, NV, USA) and by comparison with color disks from the “Roast Color Classification System” (Agtron/SCAA, Reno, NV, USA; 1995).

In order to determine the qualitative properties of coffee beans, samples were ground using a coffee grinder (KG79, De’Longhi Appliances S.r.l, Treviso, Italy) and then passed through a 30-mesh sieve.

A total of 525 coffee samples (75 samples for each group) were prepared for the HSI data acquisition. Each sample was composed of about 30 grains of coffee beans in a glass dish. All the measurements were carried out in a room at a constant temperature of approximately 25 °C and a relative humidity of 40–55%.

When modeling, all 525 samples were divided into a calibration set and prediction set by the ratio of 2:1. To avoid bias in the subset partition, the Kennard–Stone (K–S) algorithm was implemented to divide all the samples into a calibration set with 350 coffee beans and a prediction set with 175 samples [19].

### 2.2. Qualitative Properties Analysis of the Coffee Bean Samples

#### 2.2.1. Moisture Content Analysis

The moisture content of the samples was determined using the China National Standards (CNS) (GB 5009.3-2016). Coffee powder (2.000 g) was placed on a glass dish and dried in a drying oven at 105 °C for 6 h. After cooling to room temperature, the sample’s weight loss (WL) was measured using an electronic balance (precision, 0.1 mg) (BSA124S, Sartorius Lab Instruments GmbH & Co. KG, Göttingen, Germany). The drying step was repeated until the WL was less than 2 mg. The moisture contents were expressed as percentages (%). Each sample was analyzed in three replicates.

#### 2.2.2. Crude Fat Content Analysis

The crude fat contents of the samples were measured using the CNS (GB 5009.6-2016). Briefly, 5.000 g of each sample was refluxed with petroleum ether (30–60 °C) for 6 h using Soxhlet extraction. The extract was concentrated and then further dried in a drying oven at 105 °C for 1 h. After cooling to room temperature, the sample’s WL was measured using an electronic balance (precision, 0.1 mg) (BSA124S, Sartorius Lab Instruments GmbH & Co. KG, Göttingen, Germany). The drying step was repeated until the WL was less than 2 mg. The crude fat contents were expressed as percentages (%). Each sample was analyzed in three replicates.

#### 2.2.3. Chlorogenic Acid, Trigonelline, and Caffeine (CF) Content Analysis

The CA, TG, and CF contents were determined according to a previous method presented by Shao et al. (2016) with some modifications [20]. Briefly, 1.000 g of each sample (each degree of roast of coffee beans in triplicate) was extracted twice with 70% methanol at a 1:20 solid–liquid ratio using ultrasonic extractor (KQ5200DV, Kun Shan Ultrasonic Instruments Co., Ltd., Jiangsu, China) at 200 W for 15 min at 50 °C each time. The samples were spun in a centrifuge at 5000 rpm for 5 min. The supernatant was filtered through two layers of quantitative filter paper to obtain the extract and then filled with 70% methanol to 50 mL. After being filtered through a 0.22-μm membrane filter, the filtrates were analyzed by ultra high performance liquid chromatography (UHPLC) (1290infinityII, Agilent Technologies, Santa Clara, CA, USA) with a diode array detector and XDB-C18 (2.1 mm × 150 mm × 3.5 μm) column (Agilent Technologies, USA). The mobile phase comprised of acetonitrile (A) and 0.5% acetic acid–water (B) at a flow rate of 0.3 mL/min. The gradient condition was as follows: A/B ratio of 5/95 to 30/70 from 0 to 10 min, then linearly increased to 100/0 from 10 to 15 min. The column temperature was 40 °C, and the injection volume was 10 μL. The detector was set at 325 nm for CA, 267 nm for TG, and 276 nm for CF. The contents of CA, TG, and CF were quantified by the external standard method and expressed as g/kg.dw.

#### 2.2.4. Statistical Analysis

Data were expressed as the mean ± standard deviation (SD). Statistical analysis was performed using SPSS for Windows (version 17.0). Data were subjected to ANOVA followed by a *t*-test, and *p* < 0.05 was considered significant.

### 2.3. HSI Measurement and Analysis

#### 2.3.1. HSI Device

**Figure 1:**
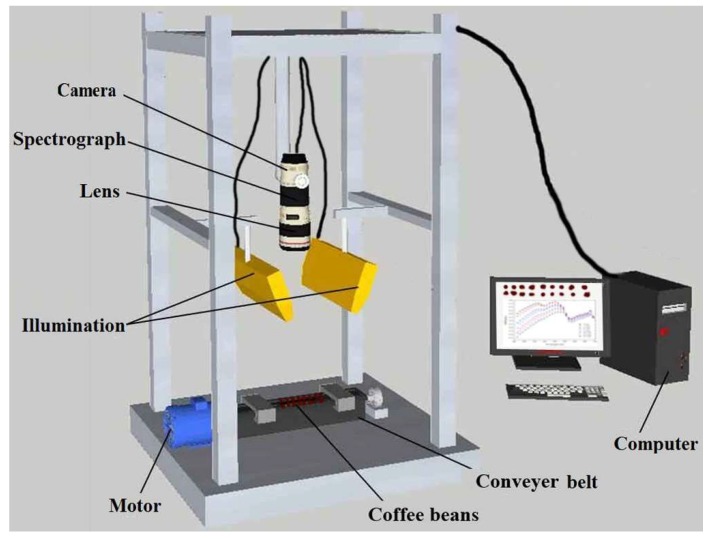
Schematic diagram of the main components of the hyperspectral imaging system.

A laboratory HSI system was established to acquire hyperspectral images of each sample in the reflectance mode, as shown in Figure 1. This HSI system consisted of an imaging spectrograph (Spectral Imaging Ltd., Specim, Finland) covered in the range of 874–1734 nm with 256 wavelengths, a specially assembled line source with two 150 W quartz tungsten halogen lamps (Fiber-Lite DC950 Illuminator, Dolan Jenner Industries Inc., Boxborough, MA, USA), a CCD camera (C8484-05, Hamamatsu, Hamamatsu City, Japan) coupled with a lens (OLES23, Specim, Spectral Imaging Ltd., Oulu, Finland), and a computer with the Spectral-Cube data acquisition software (Isuzu Optics Corp., Taiwan, China). The software was useful for setting and adjusting the parameters of the system, such as exposure time, motor speed, imaging acquisition, wavelength range, and imaging correction. Overall, all the components (except the computer) were fixed inside a dark chamber to avoid any stray light, which might affect the veracity of the HSI equipment.

#### 2.3.2. Hyperspectral Images Acquisition and Calibration

In the HSI system, the illumination and imaging spectrograph were all laid above the sample, and the two line sources focused on a linear area of the conveyer belt just below the imaging spectrograph. The imaging spectrograph was located at 320 mm above the conveyer belt. In this experiment, the exposure time was 3 ms, and the motor speed was 26 mm/s. Before sample scanning, two reference reflectance panels were adopted for white–dark reflectance correction of the sample. Then, coffee bean samples were scanned line by line on the conveyer belt to build a hyperspectral image called a “hypercube” with a dimension of (*x*, *y*, *λ*). The *x* axis is the direction of the conveyer belt movement, the *y* axis is the vertical direction of the conveyer belt movement, and *λ* axis is the wavelength in the range of 874–1734 nm. The acquired hyperspectral images were stored in the computer with a “raw” format (.BIL) before being processed. For each coffee bean sample, a hypercube was acquired by using the imaging spectrograph of ImSpector N17E (Spectral Imaging Ltd., Oulu, Finland).

To obtain relative reflectance attributes of the hyperspectral image, it was necessary to correct the raw hyperspectral images (*I_raw_*) from the dark current effect of the camera using two extra images: dark and standard white reference. A white reference (*I_white_*) image (~99.9% reflectance) was acquired from a white reference ceramic tile, and a dark reference (*I_dark_*) image (~0% reflectance) was obtained with the light source off and the camera lens thoroughly covered with its opaque cap [21]. These two reference images were then used to calculate a relative reflectance hyperspectral image (*R*) of the sample using the following equation:

(1) $$R=\frac{I_{raw}-I_{dark}}{I_{white}-I_{dark}}$$

Then, the corrected images were used as the basis for subsequent analysis, including the extraction of spectral information, the selection of relevant wavelengths, and the establishment of discrimination models [22,23]. 

#### 2.3.3. Extraction of Spectra

After the hyperspectral image calibration, the spatial structure information of the coffee bean was selected based on the region of interest (ROI). In detail, the whole coffee bean was identified as a ROI, which was manually extracted based on the initial shape of the coffee bean. Then, average spectra of the coffee samples were calculated with all the pixels in the ROI. Spectral data were extracted by the ROI function of ENVI software [24]. According to this procedure, a total of 525 mean reflectance spectra were obtained from the hyperspectral images of the coffee samples with seven roasting degrees. Each sample was acquired with 256 wavelengths in the range of 874–1734 nm, and a 525 × 256 (samples × wavelengths) matrix was formed. The above spectra extraction methods were operated with ENVI 4.6 software (ITT visual information solutions, Boulder, CO, USA).

Because of the response of the CCD detector and strong noise [12], the reflectance in two regions of 874–930 nm and 1700–1734 nm was rather low and littered. Therefore, the hyperspectral images were resized to the spectral range of 930–1700 nm with a total of 229 wavebands, resulting in a matrix with 525 × 229 (samples × wavelengths).

#### 2.3.4. Spectral Data Analytical Methods

Principal components analysis (PCA) is an unsupervised technique and has a wide application in reducing the dimension of multivariate data sets. The principle and application of PCA can be found in Bakshi (1998) [25]. Meanwhile, the score plot of principal components (PCs) is used to reveal the features of variable distribution, and the loading plot of PCs can exhibit the importance of different variables.

The random frog (RF) methodology, a novel and efficient technique for variable selection, was carried out to select important wavelengths. The RF approach borrows the framework of reversible jump Markov Chain Monte Carlo (RJMCMC) methods, and it is employed to perform feature extraction for selecting a series of variables, which describe the correlation between the predictor variables and the response variables. The execution process of the RF algorithm simulates the beating, foraging behavior of a group of frogs (solution) in a wetland (solution space). It works in an iterative manner and usually includes three steps: (1) the parameters and initialization of RF; (2) a probability-guided model searching in RF; and (3) computing the selection probability of each variable. In the interior of the RF algorithm, partial least squares regression (PLSR) is viewed as a modeling method. X (*n* × *p*) stands for the spectral matrix consisting of n samples in rows and p variables in columns. Y (*n* × 1) denotes the property of interest. Before running the RF algorithm, five parameters—*T*: number of iterations, default *T* = 10000; *Q*: number of variables in the initialized variable set, default *Q* = 50; *θ*: control variance of a normal distribution, default *θ* = 0.3; *ω*: a coefficient, default *ω* = 3; and *η*: the upper bound of the probability, default *η* = 1—should be assigned with proper values. Details of the RF methodology could be found in Li et al. (2012) [26]. As Monte Carlo strategy is embedded in the RF algorithm, the selection probability of variables is unable to be reproduced exactly. Generally, RF is implemented a couple of times (depending on the data) to minimize the influence of this random factor.

The successive projections algorithm (SPA) is a novel variable selection algorithm designed to solve collinearity problems by selecting variables with minimal redundancy [27]. However, the SPA selects relevant wavelengths through the effect analysis of independent variables on dependent variables, so the 525 × 229 matrix was set as the independent variable (X) and a 525 × 1 matrix representing the spectral responses (dummy numbers) of the 525 samples was set as the dependent variable (Y).

Least squares support vector machine (LS-SVM) was used to develop determination models for the different roasting degrees of the coffee beans [28,29]. Commonly, radial basis function (RBF) is used to establish the LS-SVM model, and the RBF kernel function can express the relationship between independent variables and dependent variables. In the modeling of LS-SVM, the scope of *γ* was set from 1 to 1000, and the optimal value of *γ* was detected by pointwise searching. Then, the full spectrum and features extracted from the two feature extraction methods were respectively set as independent variable (*X*) to develop determination models for the coffee samples with multivariate calibration. 

“The Unscrambler X 10.1” (CAMO PROCESS AS, Oslo, Norway) software and programs developed in the MATLAB R2009a (The MathWorks, Inc., Natick, MA, USA) were used to execute the multivariate data calibration.

## 3. Results and Discussion

### 3.1. Changes in Moisture, Crude Fat, TG, CA, and CF Contents of the Seven Roasting Degrees of the Coffee Beans

**Figure 2:**
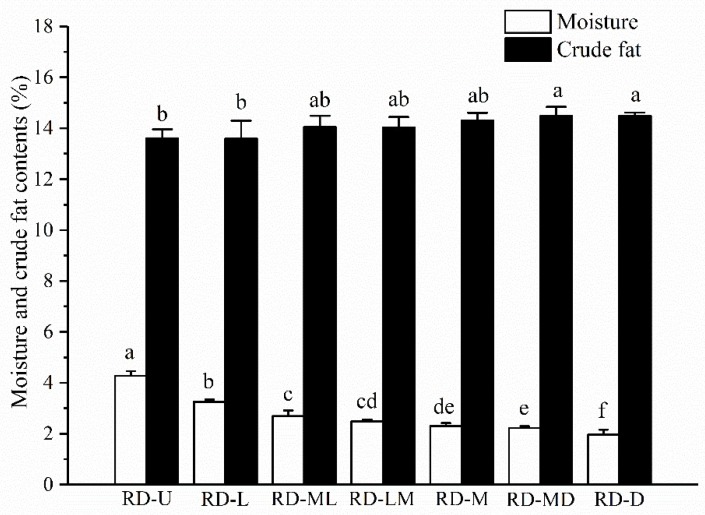
The moisture and crude fat contents of the seven roasting degrees of coffee beans. Different lowercase letters show significant differences among the groups at *P* < 0.05. The vertical bars represent means ± SD, *n* = 3. RD-U: unroasted; RD-L: light; RD-ML: moderately light; RD-LM: light medium; RD-M: medium; RD-MD: moderately dark; and RD-D: dark.

It can be observed in Figure 2 that the moisture decreased modestly with increased roasting degrees. Crude fat, a main component of coffee beans, is responsible for the aroma of coffee and has the effects of protecting volatile substances and preventing the loss of flavor during the roasting process. In this study, the longer the roasting time for the coffee beans, the slightly higher the crude fat content. This trend was consistent with previous studies [30].

**Figure 3:**
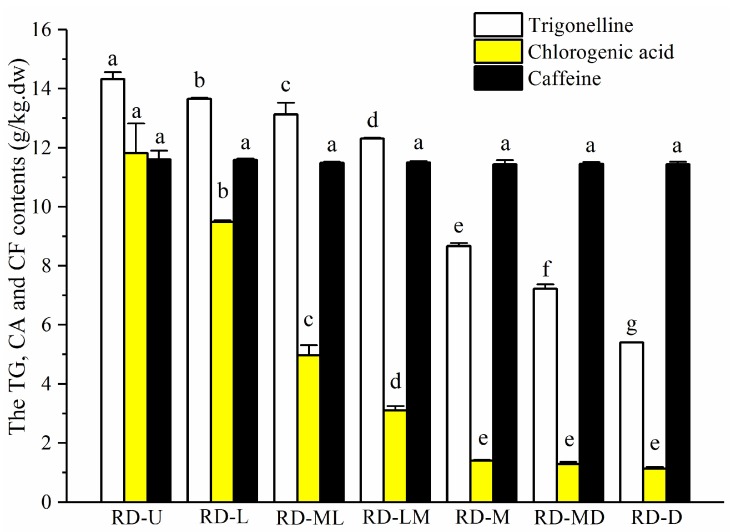
The trigonelline (TG), chlorogenic acid (CA), and caffeine (CF) contents of the seven roasting degrees of the coffee beans. Different lowercase letters show significant differences among the groups at *P* < 0.05. The vertical bars represent means ± SD, *n* = 3. RD-U: unroasted; RD-L: light; RD-ML: moderately light; RD-LM: light medium; RD-M: medium; RD-MD: moderately dark; and RD-D: dark.

There was a clear distinction in the TG and CA contents among the different roasting degrees of the coffee beans, as shown in Figure 3. During the roasting process, the contents of TG and CA decreased with the increase of the roasting degrees. TG is known to be responsible indirectly for the formation of appreciated flavor products during the roasting process [31]. CA, one of the most abundant phenolic compounds in coffee beans, exhibits a variety of pharmacologic activities [32]. It also contributes to coffee pigmentation and astringency [33]. CF is a central nervous system stimulant of the methylxanthine class, and it imparts the characteristic bitter taste to coffee [34].

Thus, the roasting method plays an important role in the quality, nutrition, and flavor of the final coffee product due to changes in the crude fat, CA, TG, and CF contents, which may be influenced by inhomogeneous roasting degrees of the coffee beans. For this reason, it is essential to develop a reliable, rapid, and nondestructive method for separating defective beans from optimum beans in the production of coffee brew, thereby reducing undesirable quality.

### 3.2. Spectral Features of the Seven Roasting Degrees of the Coffee Beans

**Figure 4:**
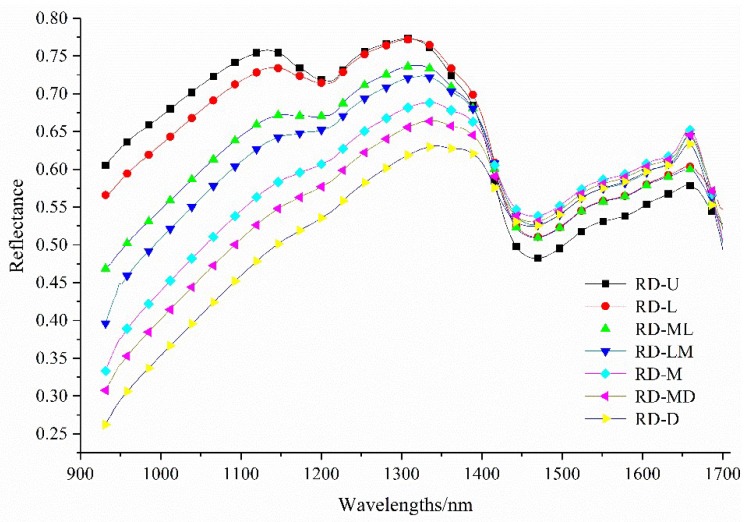
The mean reflectance spectra of the seven roasting degrees of the coffee beans. RD-U: unroasted; RD-L: light; RD-ML: moderately light; RD-LM: light medium; RD-M: medium; RD-MD: moderately dark; and RD-D: dark.

The mean reflectance spectra values of the coffee beans at seven roasting degrees are illustrated in Figure 4. It can be found that there was a similar trend among the seven roasting degrees in terms of the spectral reflectance values. However, an obvious difference in 930–1350 nm reflectance values of the coffee samples was observed.

Firstly, the reflectance values from 930 nm rose gradually. Then, a small valley around 1210 nm and a sharp dropping characteristic from 1350 nm were observed. After that, the reflectance values at 1450 nm declined drastically to the lowest levels. The bands at approximately 1210 nm and 1350 nm were related to the second overtone of C–H and the stretching of the -CH2 groups, respectively [35]. The existence of water in the coffee beans showed a feature wavelength around 1450 nm (the first overtone of the O–H stretching) [36]. Moreover, the valley around 1210 nm has been reported to be associated with the crude fat of the coffee beans [35]. The reflectance value at approximately this wavelength showed a similar tendency to the crude fat content (Figure 2), indicating that the wavelength of 1210 nm could be used for predicting the crude fat of coffee beans with different roasting degrees.

### 3.3. Principal Component Analysis of Spectral Data

**Figure 5:**
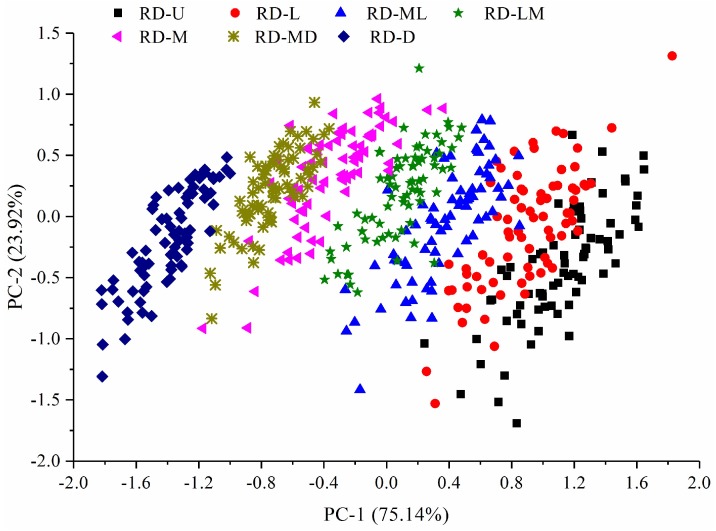
The score plot of first two principal components (PCs) of the seven roasting degrees of the coffee beans. RD-U: unroasted; RD-L: light; RD-ML: moderately light; RD-LM: light medium; RD-M: medium; RD-MD: moderately dark; and RD-D: dark.

PCA, a chemometric method for reducing the redundancy of data to achieve the purpose of compressing data, was carried out to obtain major PCs on the spectral data and display the variation among the seven roasting degrees of the coffee beans. The first two PCs (PC-1 as *x*-axis and PC-2 as *y*-axis) could explain 99.06% of the raw information, and their score plot is shown in Figure 5. The different roasting degrees of the coffee beans were mainly clustered in the side of PC-1. The PCs’ plot could expose the clustering of varieties from multiple wavebands qualitatively and achieve an obvious differentiation among the samples.

Although some differences could be observed in Figure 4 and Figure 5, there were no single or multiple wavebands performing perfect separation of both groups. Chemometric tools, therefore, were employed to extract and concentrate the spectral information [37].

### 3.4. Selection of Effective Wavelengths

**Figure 6:**
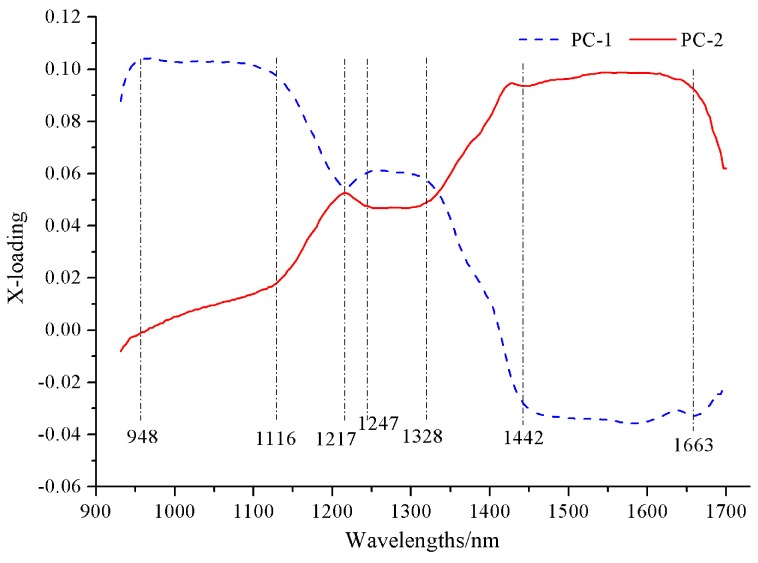
The X-loading plots of first two PCs of the principal component analysis (PCA).

The selection of optimal wavelengths was essential to reducing the redundant information in the hyperspectral images and designing an optimized multispectral imaging inspection system. To explore the spectral attributes, the X-loading of the PCs was adopted to select the wavelengths for discriminating between the roasting degrees of the coffee beans, and the X-loading plots of the first two PCs (from PC-1 and PC-2) are shown in Figure 6. A variable is of great importance when its absolute value of the X-loading is large [38]. So, seven wavelengths at 948, 1116, 1217, 1247, 1328, 1442, and 1663 nm were selected from the X-loading analysis as the alternative effective wavelengths (EWs) for discriminating between the roasting degrees of the coffee beans, as shown in Figure 6.

**Figure 7:**
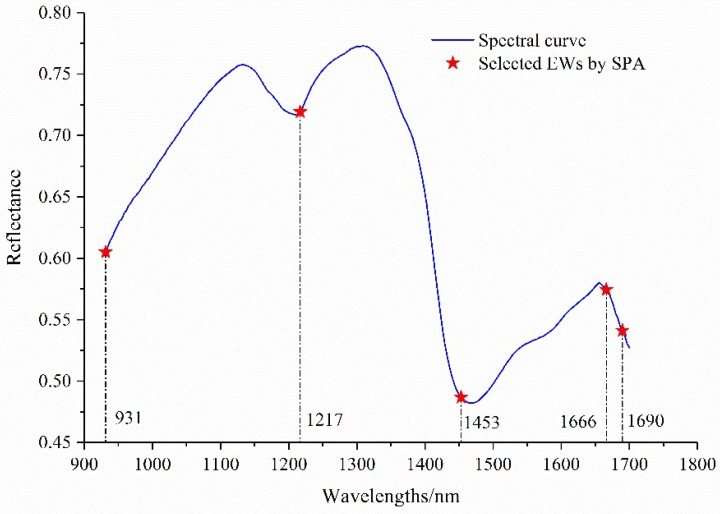
The distribution of the obtained effective wavelengths (EWs) selected by the successive projections algorithm (SPA).

Based on the spectral reflectance data matrix with 525 × 229 (samples × wavelengths) and the corresponding labeled classes of each roasting degree sample, five EWs could be identified using SPA, and the distribution of the selected EWs are shown in Figure 7. The obtained wavelengths were 1666, 931, 1217, 1453, and 1690 nm. The order of these wavelengths also showed the important relationship with the chemical components of the coffee samples.

Next, the RF methodology was adopted for effective wavelength selection. As Monte Carlo strategy was embedded in the RF algorithm, the selection probability of variables was unable to be reproduced exactly. Generally, the RF algorithm needed to be implemented a couple of times (depending on the data) to minimize the influence of this random factor. 

**Figure 8:**
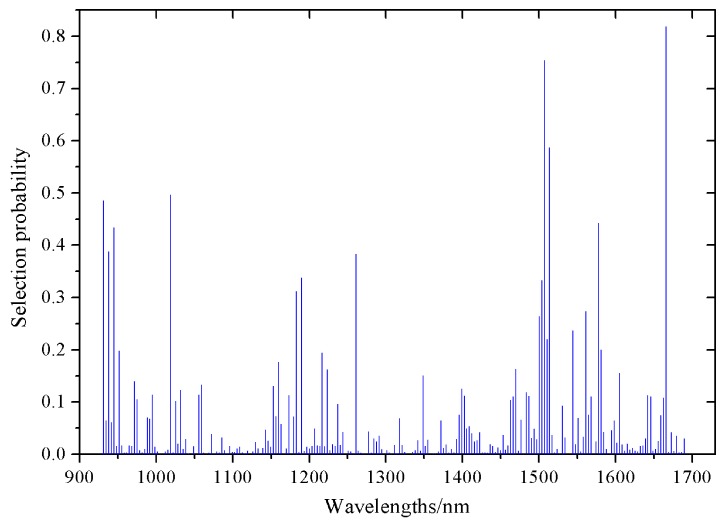
Selection probability (SP) of each wavelength averaged over 50 runs of the random frog (RF) algorithm.

Herein, RF was executed 50 times and the average value over these 50 runs was taken as the criterion for estimating the importance of each variable. The selection probability (SP) of each wavelength is shown in Figure 8. The SP values were relatively low at most wavelengths, whereas the SP of a small number of wavelengths showed relatively high SP values. This indicated that the majority of wavelengths exhibited a weak relevance for differentiating between the roasting degree of the coffee beans by HSI. All the variables were ranked in descending order according to SP. As a result, eight optimal wavelengths were selected, including 931, 945, 1507, 1183, 1018, 1261, 1656, and 1224 nm in the order of importance.

To investigate the influence of the number of wavelengths in the model, as well as to seek an optimal number of variables, the different wavelengths for differentiating between the roasting degrees of the coffee beans are collected in Table 2.

The selected effective wavelengths were mainly scattered in several regions (around 940, 1220, 1450, and 1660 nm). The effective wavelengths at 931, 945, and 948 nm were principally clustered in 940 nm, which were similarly assigned to the third overtone of the C–H stretching in the carboxylic acid [39]. The effective wavelength at 1018 nm could belong to the second overtone of O–H stretching (980 nm). The effective wavelengths at 1183, 1217, 1224, and 1247 nm were largely distributed around the regions of 1220 nm, which were similarly related to the second overtone of the C–H stretching [39]. The selected effective wavelengths at 1442, 1453, and 1507 nm were related to the first overtone of the O–H stretching around 1450 nm. The selected effective wavelengths at 1656, 1663, and 1690 nm were close to the first overtone of the C–H stretching near 1710 nm [39]. 

### 3.5. Multivariate Statistical Analysis

It is essential to select a proper calibration method in spectral analysis, so the algorithms of LS-SVM were adopted to build the discriminative models. The EWs selected by the above extraction methods were respectively set as the independent variable (*X*) for developing the LS-SVM discrimination models. Then, the results of these discrimination models summarized in a confusion matrix and overall accuracy are listed in Table 3. The numbers of correctly classified samples are listed on the diagonal, and the off-diagonal are the misclassifications.

Based on Table 3, only 18, 20, and 17 out of 175 samples in the prediction set were wrongly identified as other roasting degrees of the coffee beans using the methods of X-loading, SPA, and RF, respectively. The misclassified coffee beans mainly occurred with regards to the moderately light degree and the medium degree, which might be considered as adjacent periods with similar roasting degrees. Moreover, among the three methods for selecting the EWs, most of the coffee samples were correctly identified by their roasting degrees, resulting in an overall correct classification accuracy of 89.80% (157 vs. 175) for X-loading, 88.70% (155 vs. 175) for SPA, and 90.30% (158 vs. 175) for RF. Furthermore, 88.29% of samples could be correctly classified in the internal validation (10-fold cross-validation). The results strongly indicate that those EWs had a robust discrimination power for distinguishing between coffee beans at seven roasting degrees. The results also suggest that NIR hyperspectral imaging combined with chemometric methods have the potential to differentiate between the roasting degrees of the coffee beans.

## 4. Conclusions

The results obtained in this study provided an overview of the qualitative changes on the contents of moisture, crude fat, trigonelline, chlorogenic acid, and caffeine during the coffee roasting process (expressed as seven roasting degrees: unroasted, light, moderately light, light medium, medium, moderately dark, and dark) that were affected by the roasting conditions. The hyperspectral imaging system covering the spectra range of 874–1734 nm was used to evaluate the roasting degrees of the coffee beans, and an obvious difference in the reflectance values was observed within 930–1350 nm. X-loading analysis showed that seven wavelengths could be selected as the alternative effective wavelengths for differentiating between the different roasting degrees of the coffee beans. Principal components analysis, the random frog methodology, and the successive projections algorithm were employed to select the most suitable optimal wavelengths, based on which the least squares support vector machine discriminative models were established. In the LS-SVM model, the optimal wavelengths selected by RF showed the best results, including an overall correct classification accuracy of 90.30%. Above all, the HSI technology could provide some valuable information for the optimization of coffee bean roasting conditions and might be used for the development of portable sensors to nondestructively monitor the roasting process of coffee beans in real-time.

In further research, it is essential to investigate the relationship between the hyperspectral data and the properties of coffee beans by using some chemometric methods. In addition, HSI of coffee beans with more types of roasting conditions and more types of coffee beans should be taken into account to establish more robust models for increasing the classification accuracy. 

## Figures and Tables

**Table 1:** Roasting degrees (RDs) for coffee beans.

Groups	Roasting Degrees	Disk Scores	Roasting Time (min)	Final Temperature (°C)
RD-U	Unroasted	100	0	25.4
RD-L	Light	85	6	168.7
^1^ RD-ML	Moderately Light	75	9.5	202.3
RD-LM	Light Medium	65	10.5	219.7
RD-M	Medium	55	11.5	226.7
RD-MD	Moderately Dark	45	12.5	230.5
RD-D	Dark	35	13.5	233.1

^1^ First crack (popping) occurred at RD-ML (Moderately Light).

**Table 2:** The effective wavelengths selected by X-loading, SPA, and RF for differentiating between the roasting degrees of the coffee beans.

Methods	Effective Wavelengths (nm)
X-loading	948, 1116, 1217, 1247, 1328, 1442, 1663
SPA	931, 1217, 1453, 1666, 1690
RF	931, 945, 1018, 1183, 1224, 1261, 1507, 1656

**Table 3:** Results of least squares support vector machine (LS-SVM) determinative models for differentiating between the roasting degrees of the coffee beans in the prediction set using different methods for selecting the EWs.

Methods and Selected EWs	Roasting Degrees	RD-U	RD-L	RD-ML	RD-LM	RD-M	RD-MD	RD-D	Overall Accuracy
X-loading	RD-U	5	3	0	0	0	0	0	89.80%
	RD-L	2	15	0	0	0	0	0	
	RD-ML	0	0	20	5	0	0	0	
	RD-LM	0	0	0	28	0	0	0	
	RD-M	0	0	0	1	24	4	0	
	RD-MD	0	0	0	0	2	32	1	
	RD-D	0	0	0	0	0	0	33	
SPA	RD-U	7	1	0	0	0	0	0	88.70%
	RD-L	2	15	0	0	0	0	0	
	RD-ML	0	0	16	9	0	0	0	
	RD-LM	0	1	0	27	0	0	0	
	RD-M	0	0	0	0	24	5	0	
	RD-MD	0	0	0	1	1	33	0	
	RD-D	0	0	0	0	0	0	33	
RF	RD-U	8	0	0	0	0	0	0	90.30%
	RD-L	0	17	0	0	0	0	0	
	RD-ML	0	0	17	8	0	0	0	
	RD-LM	0	0	0	28	0	0	0	
	RD-M	0	0	0	0	22	7	0	
	RD-MD	0	0	0	1	1	33	0	
	RD-D	0	0	0	0	0	0	33	

RD-U: unroasted; RD-L: light; RD-ML: moderately light; RD-LM: light medium; RD-M: medium; RD-MD: moderately dark; and RD-D: dark.
